# LL-37 selectively targets *Plasmodium*-infected erythrocytes and exhibits antimalarial activity

**DOI:** 10.1371/journal.ppat.1014062

**Published:** 2026-03-17

**Authors:** Xiaoqin He, Yutong Zhang, Junchao Lou, Jingyao Wu, Sui Xu, Guoding Zhu, Jianxia Tang, Yaqun Fang, Jun Cao

**Affiliations:** 1 National Health Commission Key Laboratory of Parasitic Disease Control and Prevention, Jiangsu Provincial Key Laboratory on Parasite and Vector Control Technology, Jiangsu Institute of Parasitic Diseases, Wuxi, China; 2 State Key Laboratory of Genetic Evolution and Animal Models, Kunming Institute of Zoology, Chinese Academy of Sciences, Kunming, Yunnan, China; University of Manchester, UNITED KINGDOM OF GREAT BRITAIN AND NORTHERN IRELAND

## Abstract

Malaria control is challenged by the emergence of resistance to virtually all antimalarial drugs, from the frontline artemisinin to other classes, highlighting the critical need for new therapies. This study demonstrates that the human antimicrobial peptide LL-37 exhibits antiplasmodial activity against both drug-sensitive and drug-resistant parasites *in vitro*. LL-37 selectively targets infected red blood cells through membrane disruption mediated by phosphatidylserine externalization and cholesterol depletion. Elevated plasma LL-37/CRAMP levels were observed in malaria patients and infected mice, and exogenous LL-37/CRAMP administration reduced parasitemia, improved survival, and modulated pro-inflammatory cytokine levels in a mouse model. CRAMP-deficient mice showed higher susceptibility to infection, underscoring its role in host defense. Our findings reveal a naturally occurring host defense mechanism centered on LL-37/CRAMP, which acts through direct targeting of the infected erythrocyte membrane. However, therapeutic administration after infection establishment showed limited efficacy, likely due to rapid peptide degradation *in vivo*, and the effective concentrations required for direct killing *in vitro* are substantially higher than endogenous systemic levels. The reduction in systemic cytokines observed in treated mice is likely primarily attributable to decreased parasite burden rather than direct immunomodulation. Further studies are needed to evaluate stabilized analogs, optimized delivery strategies, and combination approaches before therapeutic applications can be considered.

## Author summary

Malaria remains a life-threatening disease, with rising drug resistance undermining current treatments. Host defense peptides like LL-37 offer promising alternatives, but their anti-malarial mechanisms and safety profiles are not fully understood. Here, we show that LL-37 selectively kills malaria parasite-infected red blood cells (iRBCs) by targeting two key membrane alterations caused by *Plasmodium* infection: phosphatidylserine (PS) externalization and cholesterol depletion. These changes make iRBCs vulnerable to LL-37-mediated lysis, while uninfected RBCs remain protected. We validate this mechanism by reversing the alterations—replenishing cholesterol or blocking PS with DOTAP—both of which reduce LL-37’s anti-parasitic activity. Importantly, LL-37 and its mouse homolog CRAMP exhibit minimal toxicity to uninfected cells *in vitro* and *in vivo*, with no detectable hemolysis in treated mice. In a malaria mouse model, LL-37/CRAMP suppress parasitemia and reduce disease severity. Our findings reveal a novel, host-friendly mechanism for LL-37’s anti-malarial action, highlighting its potential as a targeted therapy that bypasses drug-resistance pathways, offering new hope for combating drug-resistant malaria.

## Introduction

Malaria remains one of the most devastating infectious diseases globally. In 2024, the World Health Organization (WHO) estimated that there were 282 million malaria cases, marking an increase of 9 million cases compared to 2023 [1]. *Plasmodium falciparum* is responsible for over 90% of malaria-related deaths, with its ability to develop resistance to frontline therapies posing a critical challenge. The emergence of partial artemisinin resistance in Southeast Asia and Africa [2–4] highlights that resistance has developed to nearly all conventional antimalarial drugs, though artemisinin-based combination therapies (ACTs) remain the first-line treatment in most regions. This crisis emphasizes the urgent need for novel agents with mechanisms of action distinct from existing therapies.

Antimicrobial peptides (AMPs), which are evolutionarily conserved components of the innate immune system, show potential as alternative agents [5,6], though further preclinical and clinical data are needed to validate their utility. Their broad-spectrum activity against pathogens, including parasites, makes them attractive candidates for the development of new treatments. Cathelicidins represent a major family of antimicrobial peptides, renowned for their potent broad-spectrum activity against bacteria, fungi, and viruses [7,8]. Leucine-leucine-37 (LL-37) is the only cathelicidin expressed in humans [9]. Mice also express only one *cathelicidin* gene, denoted as cathelicidin-related antimicrobial peptide (CRAMP) [10]. Cathelicidins exert their antimicrobial effects by either disrupting microbial membranes [11] or modulating host immune responses [12,13]. Emerging evidence indicates that cathelicidins or their derivatives also target parasitic pathogens such as *Leishmania* [14], *Entamoeba histolytica* [15], *Blastocystis hominis* [16], and *Trichomonas vaginalis* [17]. However, its potential as an anti-plasmodial agent, especially against drug-resistant *P. falciparum*, remains relatively underexplored.

One of the defining characteristics of *P. falciparum*-infected red blood cells (iRBCs) is the alteration in their membrane composition. This is characterized by a reduction in cholesterol [18,19] and the externalization of phosphatidylserine (PS) [20,21]. PS externalization contributes to immune evasion by polarizing host phagocyte responses toward an immunomodulatory, anti-inflammatory phenotype [20,22]. They may also render infected red blood cells (iRBCs) more susceptible to membrane-disrupting agents. Given that LL-37 has a propensity to target anionic phospholipids, such as PS, in host cells [23,24], it is hypothesized that LL-37 could exploit the membrane abnormalities in iRBCs to exert antiplasmodial effects. The combined effect of PS externalization and cholesterol depletion disrupts the integrity of lipid rafts and reduces membrane stiffness [18,25]. Notably, unlike conventional antimalarials that target parasite metabolism or heme detoxification, membrane disruption by AMPs may avoid resistance mechanisms associated with drug efflux pumps or protease mutations.

There is growing evidence that endogenous LL-37 levels are dynamically regulated during infections. For instance, serum LL-37 levels in infants hospitalized with bronchiolitis negatively correlate with severity in infants infected by respiratory syncytial virus (RSV) but positively correlate with rhinovirus (RV) infection [26]**.** While LL-37 levels increase in infants with bacterial pneumonia, they decline as the disease progresses [27]. In malaria, however, the relationship between circulating LL-37 and disease progression remains unclear. LL-37 exhibits potential antimalarial activity, demonstrated by its ability to lyse cholesterol-depleted red blood cells (RBCs) (mimicking *Plasmodium*-infected cells) [18] and to disrupt infected RBCs, thereby reducing parasite density in infected mice [28]. However, its precise mechanism of action is unknown.

In this study, we aimed to determine the IC_50_ of LL-37 against drug-sensitive (3D7), artemisinin-resistant (803), and chloroquine-resistant (Dd2) *P. falciparum* strains and to compare its hemolytic activity against parasite-infected versus uninfected erythrocytes. We hypothesized that LL-37 specifically targets and lyses infected erythrocytes by their restructuring membranes, and that this mechanism is similar for both drug sensitive and resistant parasites.

Furthermore, we explored the association between the concentration of LL-37 or its murine ortholog CRAMP and malaria infection, and assessed the therapeutic potential of LL-37 and CRAMP in a mouse model of malaria. Utilizing gene knockout approaches, we aimed to confirm their direct antimalarial activity. Within this model, we further analyzed the relationship between parasite suppression and cytokine modulation to distinguish direct parasiticidal effects from immunomodulatory contributions.

## Materials and methods

### Ethics statement

All animal experiments were approved by the Institutional Review Board and Animal Care and Use Committee of the Jiangsu Institute of Parasitic Diseases (Ethical Approval No. JIPD-2025-015). All procedures were performed in accordance with the National Guidelines for the Care and Use of Laboratory Animals in China.

### Animals

C57BL/6 wild-type mice were purchased from Vital River Laboratory Animal Technology Co. Ltd. Cramp knockout mice (*Cramp*^−/−^), generated on a C57BL/6 background, were also obtained from Vital River. These mice carry a targeted deletion of exons 3 and 4 of the Cramp gene, which encode the entire mature CRAMP peptide, resulting in a complete functional knockout [29]. All mice were housed under standard conditions with a 12-hour light/dark cycle with free access to food and water. All mice were acclimatized to the housing for 1 week before the study. All animals were anaesthetized by isoflurane inhalation (2%) and then euthanized by cervical dislocation. All efforts were made to minimize animal suffering.

### Peptides

All peptides were synthesized by GL Biochem Ltd. (Shanghai, China). The synthesized peptides were analyzed by reversed-phase high-performance liquid chromatography and mass spectrometry to confirm purity higher than 98%. All peptides are water-soluble and were dissolved in PBS to make a 20 mM stock solution. The sequences of peptides involved are as follows:

LL-37: LLGDFFRKSKEKIGKEFKRIVQRIKDFLRNLVPRTES;
Mouse CRAMP: GLLRKGGEKIGEKLKKIGQKIKNFFQKLVPQP;
LE-16: LLGDFFRKSKEKIGKE;
FR-13: FKRIVQRIKDFLR.

### Parasite strains and cells

All the parasite strains used in the study, including *P. falciparum* 3D7 (drug-sensitive, MR4 Cat. No. MRA-102), *P. falciparum* 803 (artemisinin-resistant) [30], *P. falciparum* Dd2 (chloroquine-resistant, MR4 Cat. No. MRA-156G) [31], and *P. berghei* ANKA (MR4 Cat. No. MRA-311), were stored in our laboratory. Human erythrocytes were obtained from Wuxi Blood Center.

### Plasmodium culture

Blood stages of the laboratory clones *P. falciparum* 3D7 (sensitive strain), *P. falciparum* 803 (artemisinin-resistant strain), and *P. falciparum* Dd2 (chloroquine-resistant strain) were cultured *in vitro* according to our previous method [32]. Briefly, cultures were maintained in O-positive human RBCs as host cells at 2% hematocrit and maintained in complete medium (CM) [RPMI-1640 culture medium supplemented with NaHCO_3_ (2 mg/mL), hypoxanthine (50 μg/mL), HEPES (5.96 mg/mL), Albumax II (1%), and gentamicin (40 μg/mL)]. The cultures were diluted to 1% parasitemia and 2% hematocrit in CM and incubated in a humidified atmosphere with 5% O_2_, 5% CO_2_, and 90% N_2_ at 37°C. Prior to experiments, the cultures were highly synchronized for ring stages using 5% sorbitol twice at a 40-h interval. Briefly, ring stage cultures were centrifuged and the pellet was washed once in incomplete medium (ICM, RPMI-1640 without addition albumax). Then, the pellet was resuspended in 5% sorbitol and incubated at 37°C for 10 min. After incubation, the suspension was centrifuged and the pellet was washed once and resuspended in medium.

For schizont enrichment, parasitized erythrocytes from asynchronous *Plasmodium falciparum* cultures (>5% parasitemia) were concentrated by centrifugation (800 × g, 5 min) and resuspended in 1 mL RPMI 1640. A discontinuous Percoll gradient was prepared in a 15-mL tube by layering 3 mL of 40% iso-osmotic Percoll (IOP; density 1.075 g/mL) over 3 mL of 70% IOP (density 1.118 g/mL). The cell suspension was gently layered atop the gradient and centrifuged at 1,000 × g for 20 min at room temperature (brake disengaged). Schizonts were collected from the 40%/70% interface, washed twice in complete medium, and resuspended for downstream applications.

The development of the parasite was observed using light microscopy, and parasitemia was determined by counting at least 10,000 RBCs on Giemsa’s solution-stained thin blood smears. To ensure systematic and non-overlapping counts, a standardized protocol was followed using a customized gridded eyepiece reticule. The slide was moved methodically in a unidirectional pattern (left-to-right, top-to-bottom), with all RBCs within each sequential grid field counted until the target of 10,000 RBCs per sample was reached. This approach eliminated field duplication and ensured accurate parasitemia calculation.

### Assessment of *In vitro* antimalarial activity of peptides

A 3-day inhibition assay was used to test the antimalarial activity of LL-37 and its two truncated peptides FR-13 and LE-16. Briefly, after highly synchronization of ring stages, an aliquot of parasite inoculum (200 μL) with 1% parasitemia and 2% hematocrit was added into each well of a 96-well plate followed by a series of concentrations of LL-37, FR-13 or LE-16 peptide (dissolved in PBS and diluted with RPMI 1640). The concentrations of LL-37, FR-13 or LE-16 peptide were 1, 3, 4, 5, 7, 9, 12 and 18 μM. After 72 hours of incubation, the antimalarial effects of peptides were estimated by IC_50_. Thin blood films stained with Giemsa’s solution were counted under a microscope. IC_50_ was calculated by GraphPad Prism. Three independent assays with three technical replicates each time were conducted.

### Stage-specific and time-dependent parasite inhibition assay

To analyze both stage-specific and time-dependent antimalarial activity, highly synchronized ring-stage *P. falciparum* 3D7 parasites were cultured in 48-well plates at a starting 0.5-1% parasitemia and 2% hematocrit. LL-37 (IC_50_ concentration) was added to parasites at distinct developmental stages: rings (5 hours post-invasion, hpi), trophozoites (24 hpi), and schizonts (36 hpi). At each developmental stage, parasites were exposed to the peptide for specific durations: 3, 6, 9, or 12 hours. Following peptide exposure, parasites were washed three times with complete medium (CM) and transferred into new wells to remove the peptide. Re-invaded parasites were subsequently analyzed at 50 hpi. Parasitemia at 50 hpi was diluted 1:40 with fresh human red blood cells (RBCs) and cultured for an additional 4 days, with CM as the blank control. Parasitemia levels were determined by microscopic examination of Giemsa-stained smears. Viability was calculated as (parasitemia of LL-37-treated group/ parasitemia of untreated control group) × 100%. Lysed iRBCs were excluded from counting by excluding ghost cells (translucent, shrunken morphology) in Giemsa-stained smears; only intact RBCs (infected or uninfected) were counted to ensure accuracy. All assays were performed in three independent biological replicates, each consisting of three technical replicates.

### Hemolytic activity

*In vitro*, hemolysis assays were performed using both uninfected human RBCs [32] and *P. falciparum***-**infected RBCs. For uninfected RBCs, different peptide dilutions were incubated in triplicate with 2% hematocrit RBCs in PBS at 37°C for 15, 30, and 60 min. To evaluate potential hemolytic toxicity of LL-37, C57BL/6 mice infected or not were administered LL-37 intravenously at the maximum dose used in efficacy studies (16 mg/kg/day) for 4 consecutive days. Control mice received equivalent volumes of saline. Peripheral blood was collected 24 h after the final dose. After centrifugation (1,000 × g, 10 min), supernatant absorbance at 540 nm was measured. 1% Triton X-100 and PBS served as maximum hemolysis and negative controls, respectively, with hemolysis percentage calculated as [(A540 (peptide)- A540 (PBS))/ (A540 (Triton X-100)- A540 (PBS))]×100. For infected RBCs, schizont-enriched cultures (>70% purity) were tested. To assess stage-specific activity, enriched schizonts were diluted 1:10 with fresh RBCs and incubated to allow reinvasion, yielding ~30% ring-stage parasitemia, followed by tight synchronization with 5% sorbitol. LL-37 was then applied to synchronized cultures at ring, trophozoite, and schizont stages followed by identical hemolysis quantification.

### Mechanistic Analysis of LL-37 Targeting to Infected Erythrocytes via Membrane Modulation

To investigate the mechanism by which LL-37 targets *Plasmodium falciparum*-infected erythrocytes, the membrane properties of uninfected erythrocytes were modulated to mimic the infected phenotype through PS externalization and cholesterol depletion. PS externalization was induced by incubating erythrocytes with calcium ionophore A23187 (MCE, HY-N6687) (0.125, 0.25, 0.5, 0.75 and 1 μM) in PBS containing 1 μM CaCl₂ for 3 h at 37°C [33]. Cholesterol depletion was achieved using methyl-β-cyclodextrin (MβCD; MCE, HY-101461) (1.5, 3, 4.5, 6, and 7.5 mM) in PBS for 2 h at 37°C [18,33]. For combined treatments, cells were either pre-treated with fixed MβCD (1.5 mM) followed by varying A23187, or pre-treated with varying MβCD followed by fixed A23187 (0.75 μM), with washing between steps. All treated erythrocytes were adjusted to 2% hematocrit and incubated with LL-37 (concentration of IC₅₀) for 15 min at 37°C, and hemolysis was quantified.

### Cholesterol repletion and DOTAP competition assays to validate LL-37 targeting mechanism

To further validate the roles of phosphatidylserine and cholesterol in the antiplasmodial activity of LL-37, cholesterol reconstitution experiments were performed on normally cultured parasites, along with the addition of the cationic lipid DOTAP (1,2-dioleoyl-3-trimethylammonium propane (HY-112754A, MCE)) to competitively bind externalized phosphatidylserine and interfere with LL-37 binding [34]. For cholesterol replenishment, a 20 mg/mL cholesterol solution in ethanol was mixed with a 5% (wt/vol) aqueous methyl-β-cyclodextrin (MβCD) solution: 10 µL of cholesterol solution was added to 500 µL MβCD solution, heated at 80 °C for 10 min with repeated mixing until clear, repeated four times for a total of 50 µL cholesterol, then lyophilized in a speed vacuum concentrator until a fluffy powder remained, which was stored at −20 °C [35]. Before use, the MβCD-cholesterol complex was reconstituted in 400 µL culture medium, vortexed, and filter-sterilized through a 0.22 μm syringe filter. Ring-stage parasites (0–3 h post-invasion) were treated with the MβCD-cholesterol complex at dilution ratios of 1:20, 1:40, and 1:80 in a 96-well plate for 38 h at 37 °C, then washed with incomplete culture medium (ICM) to remove the complex, resuspended in complete medium (CM), transferred to a new 96-well plate, and incubated with LL-37 at its IC_50_ for 6 h followed by smear preparation and parasitemia quantification. Membrane cholesterol levels in iRBCs post-cholesterol repletion were quantified using the Amplex Red Cholesterol Assay Kit (Beyotime, China; Catalog No.S0211S) strictly following the manufacturers’ instructions, with all samples run in triplicate. For DOTAP supplementation assays, late-stage parasites (36–40 h, ∼4% parasitemia) were treated with DOTAP at 5, 10, or 20 μM, alongside blank control, and vehicle control controls. After incubation with or without LL-37 for 6 h, parasitemia was determined from smears. DOTAP was dissolved in dimethyl sulfoxide (DMSO) to prepare a high-concentration stock solution. Working concentrations of DOTAP (including 20 μM and other test concentrations) were generated by serial dilution of the stock solution with RPMI 1640 culture medium. The vehicle control group was prepared by adding an equal volume of pure DMSO to the same culture medium, ensuring the final DMSO percentage was identical across all DOTAP treatment groups and the vehicle control (≤0.1%). Flow cytometry was used to analyze the competitive binding of LL-37 and DOTAP to PS on late-stage iRBCs. Enriched late-stage parasites were washed with pre-warmed PBS, aliquoted into tubes (400 μL per tube), and divided into five groups: Hoechst single-staining, Annexin V single-staining, negative control (N group), LL-37 (5 μM), and DOTAP (20 μM). The two single-staining groups were stained separately to establish gating parameters for flow cytometry analysis. All groups were supplemented with 4 μL Hoechst staining solution, incubated at 37°C for 5 min, washed twice with 1× Binding Buffer, and resuspended in 400 μL 1× Binding Buffer. Annexin V staining solution, DOTAP, and LL-37 were added sequentially according to grouping, incubated at 37°C for 20 min in the dark, and samples were analyzed by flow cytometry. Unbound LL-37 in the supernatant was quantified by ELISA to verify DOTAP-mediated PS binding competition. Enriched late-stage parasites were washed with pre-warmed PBS, aliquoted into tubes (400 μL per tube), and divided into four groups: LL-37 (5 μM) alone, LL-37+DOTAP (5 μM), LL-37+DOTAP (10 μM), and LL-37+DOTAP (20 μM). Pre-diluted DOTAP was added to achieve the indicated working concentrations; the LL-37 alone group received equal volume of DMSO at the same dilution ratio. All groups were supplemented with LL-37 to a final concentration of 5 μM, mixed thoroughly, incubated at 37°C for 5 min, and centrifuged at 3,000 rpm for 50 s to collect the supernatant. The concentration of LL-37 in supernatant was examined by Human LL-37 ELISA Kit (Sangon Biotech, Shanghai, China; Catalog No. D711299) following the manufacturer’s instructions. For the combined cholesterol and DOTAP treatment, ring-stage parasites (0–3 h) were pretreated with MβCD-cholesterol complex at a 1:40 dilution for 38 h, washed with ICM, resuspended in CM, then co-treated with 20 μM DOTAP (or DMSO) and LL-37 for 6 h before parasitemia assessment.

### Parasite infection and *in vivo* antimalarial assay

For *in vivo* infection, *Plasmodium berghei* ANKA (*P.b* ANKA) parasites were thawed rapidly at 37°C and injected intraperitoneally (i.p.) into donor mice. Parasites were passaged in C57BL/6J mice. Donor mice exhibiting approximately 30% parasitemia were euthanized, and blood was collected for infection of mice in the antimalarial assay.

The antimalarial activity of test peptides was evaluated using two therapeutic regimens in female C57BL/6J mice. For the prophylactic/early treatment model, the classical 4-day suppressive test was used as previously described [36]. On day 0, mice were inoculated with 1.0 × 10^6^ *P.berghei-*infected erythrocytes harvested from donor mice with the infection route differing by assessment batch: mice in the Endpoint-Assay Batch received an intravenous (i.v.) injection to achieve synchronous parasitemia for acute analysis, while mice in the Survival-Assay Batch received a standard intraperitoneal (i.p.) injection for long-term monitoring. One hour post-infection, treatment was initiated intravenously once daily for four days, administering the test peptides at 1, 4, or 16 mg/kg, chloroquine diphosphate (16 mg/kg) as a positive control, or saline as a negative control (n = 6 per group).

For the therapeutic intervention model, designed to mimic therapy after infection establishment, all mice were infected via i.p. injection. Treatment with a high dose of the test peptides (16 mg/kg), chloroquine (16 mg/kg), or saline (n = 6 per group) was administered intravenously for four consecutive days only after peripheral parasitemia reached 8–15%, with this regimen applied to separate Endpoint- and Survival-Assay Batches that were both infected via the i.p. route.

In the Endpoint-Assay Batches for both models, mice were euthanized on day 4, 24 hours after the final dose for terminal sampling. Parasitemia was evaluated from thin tail blood smears from Day 0 to Day 4. These smears were fixed with methanol, stained with 10% Giemsa for 15 min, and examined under a light microscopy (100× objective). Parasitemia was quantified by counting parasitized erythrocytes among ≥3,000 red blood cells per smear. For histopathology, liver, spleen and brain were collected immediately after euthanasia, and rinsed three times in ice-cold phosphate-buffered saline (PBS) to remove residual blood, and fixed in 4% paraformaldehyde for 24 h at 4°C. Fixed tissues were paraffin-embedded, sectioned, and stained with hematoxylin and eosin (H&E) for histological evaluation. Plasma was isolated by centrifugation at 3,500 × g for 30 min at room temperature. Cytokine concentrations (IL-1β, IL-6, IFN-γ, TNF-α) were measured in plasma using commercial ELISA kits (Dakewe, China; Catalog Nos.: 1210122, 1210602, 1210002, 1217202) according to the manufacturer’s protocols.

In the Survival-Assay Batches for both models, a minimal confirmation of parasitemia was performed on Day 4, after which mice were monitored daily for 21 days. To assess disease progression, mice were evaluated according to the rapid murine coma and behavior scale (RMCBS) for quantitative assessment of murine cerebral malaria (CM), as described [37] with minor modifications. Briefly, six parameters were used to evaluate the animals, including ruffled fur, hunched posture, wobbly gait, limb paralysis, convulsions, and coma. Each parameter was scored from 0 to 2. For example, fur condition was scored as 0 for ruffled/piloerected hair, 1 for dusty hair or hair out of place, and 2 for normal, clean, and shiny hair. In this scale, the minimum RMCBS score was 0 and the maximum score was 12. Following infection, the assessment was performed every 24 hours until 7 days post-infection (p.i.) and every 12 hours thereafter. Based on Evans blue staining confirming blood-brain barrier disruption, an RMCBS score of ≤ 6 was defined as indicating the onset of experimental cerebral malaria (ECM). Mice were euthanized when they reached a humane endpoint criterion of RMCBS scores of 6 or less, and this timepoint was defined as “end stage”. All tissue/blood samples were collected 24 hours after the final dose. Humane endpoints were strictly enforced: moribund mice exhibiting irreversible symptoms were euthanized by cervical dislocation under anesthesia (isoflurane 2%) and the time of death was recorded. All surviving mice were humanely euthanized at 21 d.p.i. for endpoint analysis.

### Detection and statistical analysis of serum LL-37 expression in human and mouse models

The study involving human subjects was conducted in accordance with relevant ethical guidelines and regulations. This study utilized existing, anonymized plasma samples obtained from the Jiangsu Malaria Biobank. The samples consisted of specimens from 63 patients with confirmed *P.falciparum*-infection and 63 age- and sex-matched healthy controls. The secondary use of these de-identified samples for research is permitted under national ethical regulations, which grant an exception to the requirement for project-specific informed consent. The operational protocols of the biobank and the specific research protocol for this study were reviewed and granted an exemption by the Ethics Committee of the Jiangsu Institute of Parasitic Diseases (Approval No. JIPD-2022–005).

Meanwhile, 12 female C57BL/6 mice (6–8 weeks old) were infected with 1.0 × 10⁶ *P.berghei* ANKA-infected erythrocytes via intraperitoneal injection alongside 12 uninfected control mice in compliance with animal care protocols. Mice were culled at day 4 post-infection (when parasitemia reached ~8–15%) for plasma collection. Following infection, mouse blood was collected via cardiac puncture under anesthesia. Human and mouse plasma samples were separated by centrifugation at 3,000 × g for 10 minutes and stored at −80°C until further analysis. For quantification of LL-37, sample concentrations were measured using species-specific commercial ELISA kits, human LL-37 ELISA Kit (Sangon Biotech (Shanghai) Co., Ltd.) and murine CRAMP ELISA Kit (Cat# CSB-E15061m, CUSABIO, USA), strictly following the manufacturer’s instructions, with all samples run in triplicate. Finally, statistical analysis was performed using GraphPad Prism software, where data were expressed as mean ± SD and median values, and group comparisons were carried out with the non-parametric Mann-Whitney U test, considering a p-value of less than 0.05 as statistically significant.

### Statistical analysis

Continuous variables were summarized as means ± standard error of the mean (SEM). Two-group comparisons used two-tailed unpaired Student’s *t*-test. Multi-group analyses employed one-way analysis of variance (ANOVA) with Dunnett’s multiple comparisons test (comparisons versus control group). Survival curves were compared by log-rank test. Statistical significance was defined as *p* < 0.05 (two-tailed). All analyses were performed using GraphPad Prism v10.0.

## Results

### 1. *In Vitro* antimalarial activity of LL-37 and analysis of its truncated peptides

**Fig 1:**
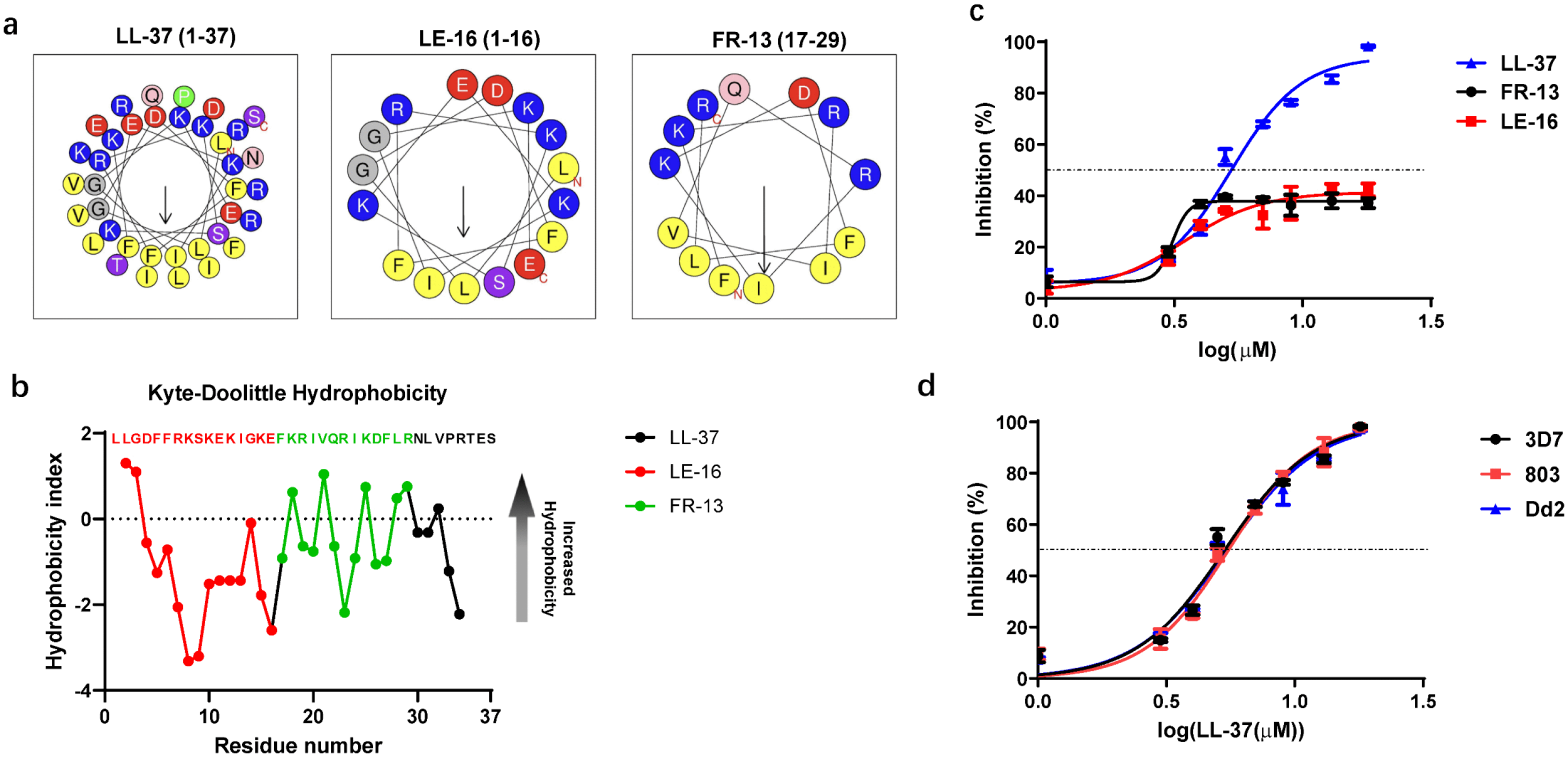
*In vitro* antimalarial activity of LL-37 and its truncated peptides. **(a)** Helical wheel analysis of antimicrobial peptide LL-37. The helical wheel diagram was generated using HeliQuest to visualize the spatial distribution of residues. Residues are color-coded by physicochemical properties: yellow (hydrophobic: I, L,V,F), blue (positively charged: K,R), purple (uncharged polar: S,T), red (negatively charged: D,E), pink (uncharged polar amide: **Q)**, green (non-polar: **P)**, gray (aliphatic: G,A). The projection angle is rotated to maximize amphipathicity with hydrophobic residues (yellow) clustered on one face and polar/charged residues (blue/red/purple) on the opposing face. **(b)** Hydrophobicity profile of antimicrobial peptide LL-37. Residue numbers are indicated on the x-axis. Hydrophobicity index was calculated using the Kyte-Doolittle scale (window size = 5). Positive values indicate hydrophobic regions, negative values indicate hydrophilic regions**. (c)** IC_50_s of LL-37 and its truncated peptide against *P. falciparum* 3D7. **(d)** IC_50_s of LL-37 against sensitive and resistant strains of *Plasmodium falciparum.* Data in c and d are presented as means ± SEM from three biological replicates.

To evaluate the antimalarial activity of LL-37 and the functional region responsible for the antimalarial activity of LL-37, two truncated peptides, the highly hydrophobic LE-16 fragment (residues 1–16) and the amphipathic α-helical FR-13 fragment (residues 17–29) (Fig 1a, 1b), were designed and evaluated alongside the full-length peptide. The efficacy against the *Plasmodium falciparum* 3D7 strain of LL-37 and its truncated peptides was assessed *in vitro*. LL-37 exhibited antimalarial activity, with a half-maximal inhibitory concentration (IC₅₀) of about 5.3 ± 0.3 μM against *P. falciparum* 3D7 strain (Fig 1c). Interestingly, at lower concentrations (below ~5 μM), all peptides, including both fragments, reached a similar partial inhibition plateau of about 40%. However, only the full-length LL-37 demonstrated a capacity to surpass this plateau at higher concentrations, achieving complete growth inhibition. Although both fragments retained partial antiplasmodial activity, their overall efficacy was markedly reduced compared to intact LL-37 (Fig 1c), suggesting that neither the C-terminal helical domain nor the N-terminal hydrophobic segment alone is sufficient to recapitulate the full activity of LL-37. The markedly enhanced potency of the full-length peptide implies that its structural integrity, likely involving cooperative interactions between domains, is critical for optimal antimalarial action. Furthermore, LL-37 displayed consistent efficacy against drug-resistant strains, with IC₅₀ values of 5.5 ± 0.3 μM for artemisinin-resistant 803 and 5.4 ± 0.3 μM for chloroquine-resistant Dd2 (Fig 1d), indicating that its mechanism remains effective across genetically distinct parasites.

### 2. LL-37 exhibits stage-specific and time-dependent anti-malarial activity

**Fig 2:**
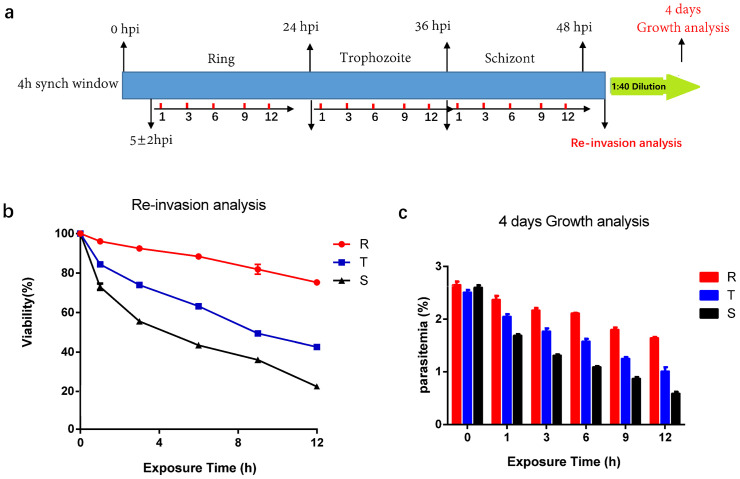
Stage-specific and time-dependent antiplasmodial activity of LL-37 in the blood stage. **(a)** Schematic of the experimental design. Highly synchronized *P. falciparum* 3D7 parasites were treated with LL-37 at its IC_50_ for 1, 3, 6, 9 and 12 h across three consecutive periods of the intraerythrocytic life cycle. Subsequently, the parasitemia of re-invasion was analyzed at 50 hpi, and another analysis was conducted after a 1:40 dilution and 4 days of culture. **(b)** Viability of parasites re-invasion at 50 hpi was measured. **(c)** Parasites were washed, followed by 1:40 dilution, and cultured for 4 days while parasitemia was measured. Data in b and c are presented as means ± SEM of 10,000 RBCs from three biological replicates.

We further evaluated the activity of LL-37 against different blood stages using highly synchronized *P. falciparum* parasites. Parasites were exposed to LL-37 at IC_50_ for different treatment times (1, 3, 6, 9, 12h) at the ring (5 hours post invasion (hpi)), trophozoite (24 hpi), and schizont (36 hpi) stages (Fig 2a). Comprehensive stage-specific and time-dependent assays revealed that LL-37 possesses significant inhibitory activity against *P. falciparum* 3D7 parasites across all major intraerythrocytic developmental stages of rings (R), trophozoites (T), and schizonts (S). Quantitative analysis of parasitemia viability at 50 hpi, following LL-37 exposure, showed a clear sensitivity level that ring stages were the least sensitive (highest viability), followed by trophozoites, with schizonts exhibiting the highest sensitivity and lowest viability (Fig 2b). Furthermore, the antimalarial activity of LL-37 displayed a pronounced time-dependent relationship within each developmental stage (Fig 2b). This time-dependent effect indicates the importance of exposure duration for achieving maximal inhibitory activity against the parasites, irrespective of its developmental phase. Also, the same results were shown in further 4 days growth analysis (Fig 2c). Collectively, LL-37’s efficacy is maximized by extended exposure and targets schizonts most potently.

### 3. LL-37 selectively targets infected erythrocytes by exploiting synergistic phosphatidylserine externalization and cholesterol depletion

**Fig 3:**
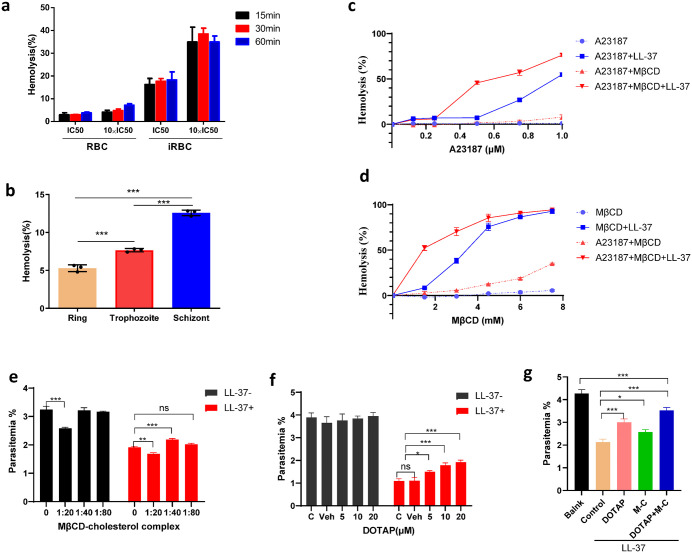
LL-37 selectively targets infected erythrocytes by exploiting synergistic phosphatidylserine externalization and cholesterol depletion. **(a)** Comparative hemolysis of uninfected erythrocytes (uRBC) and schizont-infected erythrocytes (iRBC) exposed to LL-37 at its IC_50_ and 10 × IC_50_ concentrations. **(b)** Stage-specific hemolysis of *P. falciparum*-infected erythrocytes treated with LL-37 (IC_50_). R: Ring stage; T: Trophozoite stage; S: Schizont stage. **(c)** Hemolysis of LL-37 under increasing A23187 concentrations with or without 1.5 mM MβCD. **(d)** Hemolysis of LL-37 under increasing concentrations of MβCD with or without 0.75 μM calcium ionophore A23187. **(e)** Parasite parasitemia under treatment with different proportions of MβCD-cholesterol complexes with or without LL-37 present. **(f)** Parasite parasitemia under treatment with different concentrations of DOTAP with or without LL-37 present. **(g)** Parasite parasitemia of LL-37 treatment with MβCD-cholesterol complexes and/or DOTAP. All data are presented as means ± SEM from three biological replicates. ns: not significant; *p < 0.05, *p < 0.01, ***p < 0.001 indicate statistically significant differences.

Given the heightened susceptibility of schizonts, we hypothesized that LL-37 selectively targets pathological alterations in infected erythrocytes (iRBCs). LL-37 exhibited stage-selective hemolytic activity against *P. falciparum*-infected erythrocytes while demonstrating minimal toxicity to uninfected red blood cells (uRBCs) (Fig 3a). At its IC_50_, LL-37 induced minimal hemolysis (<10%) in uRBCs, even after 60 minutes, a resistance maintained at 10 × IC_50_ (Fig 3a). In contrast, schizont-enriched infected RBCs (iRBCs) showed significant lysis of 18.3% **±** 2.0% at IC_50_, escalating to 34.9% ± 1.5% at 10 × IC_50_ (Fig 3a). Schizonts were 2.3-fold and 1.8-fold more sensitive to LL-37 than ring or trophozoite stages, respectively (Fig 3b).

To dissect this selectivity, we reconstituted iRBC-like membrane pathology in normal RBCs. Individual PS externalization (A23187) or cholesterol depletion (MβCD) dose-dependently sensitized RBCs to LL-37. For instance, 1 μM A23187 or 7.5 mM MβCD combined with IC_50_ of LL-37 induced hemolysis of 54.8% and >90%, respectively, compared to <10% by either modulator alone (Fig 3c, 3d). Crucially, combined subthreshold doses acted synergistically, adding 0.75 μM A23187 to 1.5 mM MβCD boosted LL-37-mediated hemolysis from 8.4% to 52.4%, a 43.9% increase (Fig 3d).

To definitively validate this mechanism, we investigated whether reversing these alterations could protect iRBCs. We first identified an effective rescue strategy that used an MβCD-cholesterol complex to supply cholesterol. Direct quantification of membrane cholesterol (S1a Fig) showed that treatment with 1:80, 1:40, and 1:20 MβCD-cholesterol complexes all significantly increased iRBC membrane cholesterol relative to the infected control group (*p < 0.05; ***p < 0.001), with levels rising in a dose-dependent manner (1:80 < 1:40 < 1:20). We then evaluated the protective effect of these cholesterol-replete iRBCs against LL-37. While all MβCD-cholesterol ratios increased membrane cholesterol, only the 1:40 dilution yielded optimal protection, approximately 20% restoration of iRBC survival as shown in Fig 3e. This discrepancy could potentially be explained by parasite tolerance to membrane cholesterol levels: the 1:20 dilution (highest cholesterol) likely disrupted the delicate lipid balance required for parasite development (inducing non-specific anti-parasitic toxicity, even without LL-37), while the 1:80 dilution (lowest cholesterol) failed to sufficiently reverse the LL-37-sensitive “cholesterol-depleted” membrane state. The 1:40 dilution, by contrast, restored cholesterol to a level that counteracted LL-37’s targeting (by reducing membrane vulnerability) without impairing parasite viability, thus representing the optimal balance between cholesterol repletion and parasite fitness.

In parallel, we evaluated DOTAP (a PS-binding cationic lipid) and confirmed its competition with LL-37 for PS binding. Flow cytometry analysis (S1b Fig) showed that both LL-37 and DOTAP reduced Annexin V positivity in PS-exposed iRBCs (Q2 quadrant), reflecting PS binding by these molecules (rather than reduced PS exposure). Quantification of Annexin V positivity (S1c Fig) further verified that both LL-37 and DOTAP treatment decreased PS accessibility to Annexin V. To directly assess competition, we measured LL-37 levels in supernatants post-co-incubation with DOTAP (S1d Fig). DOTAP concentration-dependently increased free LL-37 (e.g., 20 μM DOTAP raised supernatant LL-37 by ~30%), confirming reduced LL-37 binding to iRBCs. The protective effect was concentration-dependent but plateaued at higher concentrations, with 20 μM considered a saturating concentration that restored approximately 30% of the lost survival (Fig 3f). Most conclusively, the combination of the optimized 1:40 MβCD-cholesterol and saturating 20 μM DOTAP yielded a strong synergistic effect, restoring nearly 65% of the lost survival rate (Fig 3g).

Together, these results demonstrate that the membrane-specific pathology induced by *Plasmodium infection,* characterized by cholesterol depletion and phosphatidylserine externalization, is essential for the selective hemolytic activity of LL-37. Reversing these alterations effectively protects infected cells, providing direct functional evidence for the mechanism underlying LL-37’s anti-malarial action.

### 4. LL-37 expression is significantly elevated in malaria-infected hosts

**Fig 4:**
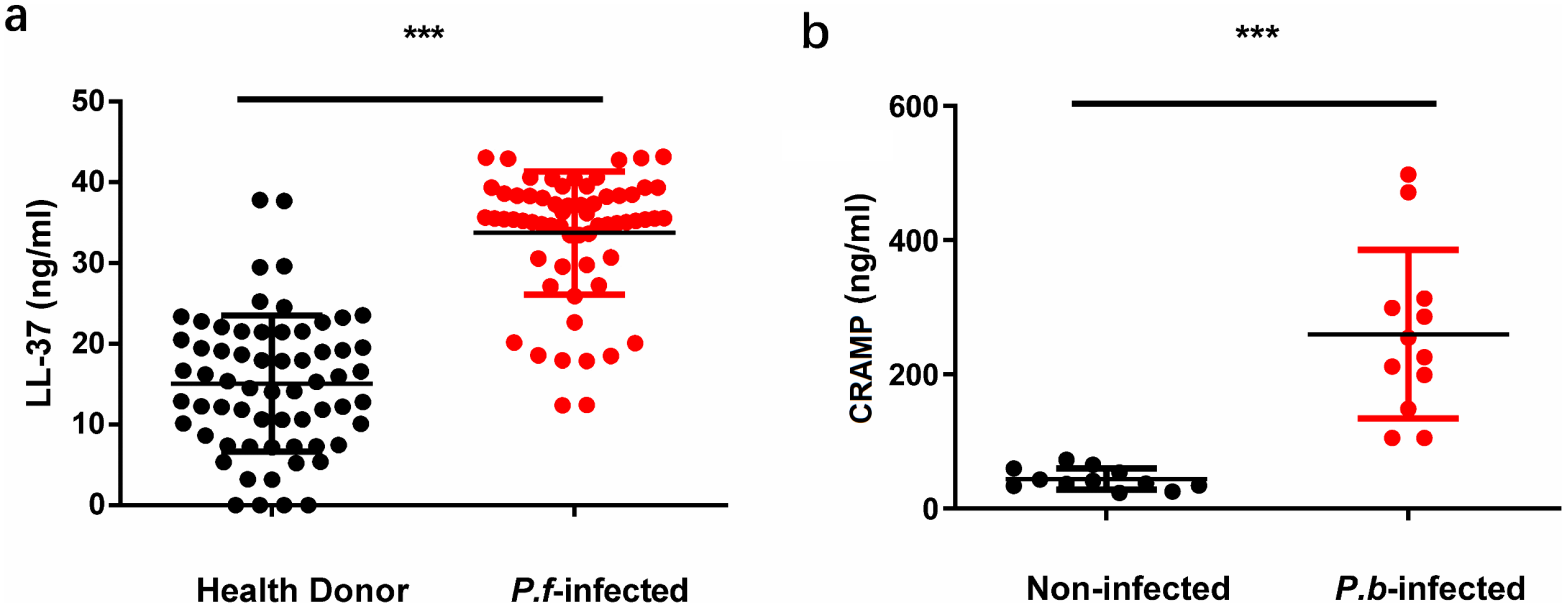
Elevated plasma levels of LL-37 and CRAMP in malaria-infected individuals and *Plasmodium*- infected mice. **(a)** Plasma levels of LL-37 in *Plasmodium falciparum* (*P.f*) - infected patients and healthy donors. **(b)** Plasma levels of CRAMP in *Plasmodium berghei* (*P.b*) - infected mice and non-infected control mice. Each dot represents an individual sample. Horizontal lines indicate the median values. ***p < 0.001 indicate statistically significant differences.

Having established LL-37’s antiplasmodial mechanism, we asked whether hosts naturally mobilize this defense during infection. Quantitative ELISA confirmed significant upregulation of serum LL-37. Quantitative ELISA analysis demonstrated significant upregulation of serum LL-37 in *Plasmodium*-infected hosts, with human malaria patients (n = 63) exhibiting 2.2-fold higher levels than healthy controls (33.7 ± 6.2 ng/mL vs 15.1 ± 9.1 ng/mL; median 35.4 vs 15.4 ng/mL, *p* < 0.0001, Mann-Whitney U test), where 92% (58/63) of patients exceeded the 30 ng/mL threshold compared to only 13% (8/63) of controls (Fig 4a). This threshold was statistically derived as the mean LL-37 level of the healthy controls plus two standard deviations, representing the upper limit of the normal range. The serum LL-37 levels observed in our healthy controls (15.1 ± 9.1 ng/mL) are consistent with previously reported ranges for normal individuals (e.g., 5–20 ng/mL), while the elevated levels in malaria patients resemble those documented in other inflammatory conditions [38]. Meanwhile, *P. berghei*-infected mice (n = 12) exhibited a 6-fold elevation in serum CRAMP relative to uninfected controls (n = 12; 262.4 ± 126.6 ng/mL vs 43.6 ± 16.3 ng/mL; median 240.4 vs 39.2 ng/mL, *p* < 0.0001, Mann-Whitney U test), with all infected animals exceeding 100 ng/mL while controls remained below 73 ng/mL, confirming species-conserved induction of this antimicrobial peptide (Fig 4b). This conserved induction indicates LL-37’s physiological role in malaria defense.

### 5. Prophylactic, but not therapeutic, administration of exogenous LL-37/CRAMP reduces malaria pathogenesis and improves host survival

**Fig 5:**
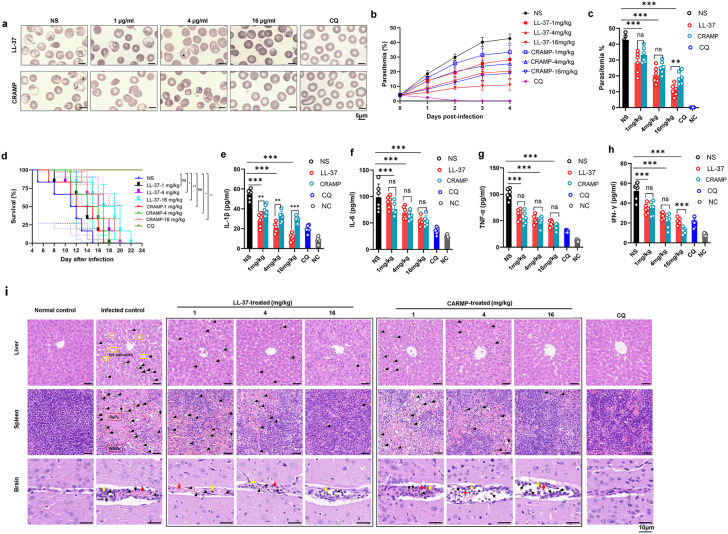
Exogenous LL-37 and CRAMP treatment confers antiplasmodial and anti-pathological effects. **(a)** Representative images of blood smears (day 4 post-infection) following treatments with different concentrations of LL-37 and CRAMP (n = 6 mice per group). **(b)** Dynamic changes in parasitemia from day 0 to day 4 post-*P. berghei* ANKA infection in untreated control mice and mice treated with LL-37/CRAMP (1, 4, 16 mg/kg/day, intravenous administration for 4 consecutive days). Parasitemia was determined by microscopic examination of Giemsa-stained thin blood smears at 24 h intervals. Data are presented as mean ± SEM (n = 6 mice per group). **(c)** Quantification of parasitemia (day 4, n = 6 mice/group) in mice treated with different concentrations of LL-37 and CRAMP. Data are presented as mean ± SEM (n = 6 mice per group). **(d)** Survival curves of mice administered different concentrations of LL-37 and CRAMP (day 4, n = 6 mice per group). Statistical analysis: Log-rank (Mantel-Cox) test, ns: not significant; *p < 0.05, **p < 0.01 vs. NS group. **(e–h)** Quantification of various inflammatory cytokines in mice treated with different concentrations of LL-37 and CRAMP (day 4, n = 6 mice per group). **(i)** Representative H&E-stained sections of liver, spleen, and brain tissues (day 4, n = 3 mice per group) from mice under distinct conditions: normal control, infected control, CQ positive control, and treatment with different concentrations of LL-37 or CRAMP. Black arrows point to malarial pigment; red arrows indicate the sequestration of RBCs; yellow arrows denote inflammatory cells; yellow boxes show fat vacuoles; red boxes indicate RePu or WhPu. Scale bar = 10 μm. ns: not significant; **p < 0.01, ***p < 0.001 indicate statistically significant differences. In all panels, NS (normal saline) served as a negative control, treated with normal saline (0.9% NaCl); NC (normal control) denotes the normal uninfected control group, serving as a baseline for normal physiological status and CQ (chloroquine) as a positive control.

To translate *in vitro* and observational findings, we evaluated the efficacy of host defense peptides LL-37 (human) and its murine ortholog CRAMP using a *P. berghei* infection model. We first assessed their activity in a prophylactic 4-day suppressive test. Intravenous administration of LL-37 and CRAMP (1–16 mg/kg/day for 4 days) induced a significant, dose-dependent suppression of day 4 parasitemia (Fig 5a, 5b). Dynamic monitoring of parasitemia from day 0 to day 4 revealed that peptide treatment effectively delayed the exponential rise of parasite load compared to the untreated control group, with the 16 mg/kg dose exhibiting the most inhibitory effect across all time points (Fig 5b). Consistent with the kinetic data, a single-time-point analysis on day 4 also confirmed dose-dependent reduction of parasitemia in peptide-treated mice (Fig 5a, 5c), which aligned with the time-course observations and validated the antiplasmodial efficacy of LL-37 and CRAMP. LL-37 and CRAMP also significantly prolonged survival days compared to saline controls, the median survival day of saline controls was 11.5, while that of 1 mg/kg/day LL-37 and CRAMP were 14 and 13.5, 4 mg/kg/day LL-37 and CRAMP were 16 and 15, 16 mg/kg/day LL-37 and CRAMP were 18 and 17 (Fig 5d). Consistent with the extended survival, progression to ECM was delayed in both LL-37- and CRAMP-treated groups (S2 Fig). To address the potential hemolytic effects of LL-37/CRAMP, we measured plasma hemoglobin levels (A540) in treated and control mice. As shown in S3 Fig, no significant differences in hemolysis were observed between LL-37/CRAMP-treated groups (16 mg/kg/day) and saline-treated control groups in both *P. berghei-*infected and uninfected mice (all *p* > 0.05). This result indicates that the therapeutic doses of LL-37/CRAMP used in our study do not cause *in vivo* hemolysis, despite a minimal 3–5% *in vitro* lysis of uninfected RBCs (Fig 3a). The discrepancy between in *vitro* and *in vivo* results suggests that the physiological microenvironment *in vivo* may protect uninfected RBCs from peptide-induced lysis.

Beyond parasite control, prophylactic treatment profoundly attenuated the malarial inflammatory response. Plasma levels of key pro-inflammatory cytokines IL-1β (Fig 5e), IL-6 (Fig 5f), TNF-α (Fig 5g), and IFN-γ (Fig 5h) measured on day 4 were significantly reduced compared to controls. Notably, the reduction in pro-inflammatory cytokines (IL-1β, IL-6, TNF-α, IFN-γ) observed in LL-37/CRAMP-treated mice (Fig 5e-5h) coincided with significantly lower parasitemia in these animals (Fig 5a,b). Therefore, it is likely that the decreased systemic cytokine levels are primarily attributable to the reduced parasite burden, which diminishes the antigenic stimulus for immune activation. Furthermore, histopathological analysis revealed a reduction of organ pathology (Fig 5i).

Among all treatment groups, the groups treated with 16 mg/kg LL-37 and CRAMP exhibited the greatest protective capacity for the tissues, including liver, spleen and brain, the therapeutic effect was equivalent to that of the chloroquine control group (Fig 5i). For liver, the hepatic lobule structure in the untreated group exhibited severe damage, the hepatic plates were not closely arranged. The liver contained a large number of fat vacuoles and hemozoin deposition. The groups from LL-37 and CRAMP-treated mice exhibited markedly reduced hemozoin deposition, and restored the normal structure of the hepatic lobules with a tighter arrangement between hepatocytes. While spleen sections showed a significantly widened red pulp (RePu) region and atrophy in the white pulp (WhPu) area in the untreated group, indicating severe anemia and more extramedullary hematopoiesis. The groups treated by LL-37 and CRAMP restored the WhPu area indicating improvement of anemia. Another indicator of the improvement of the spleen tissue in the treatment groups was a significant reduction in the deposition of malaria pigment (Fig 5i). In brain sections, the adhesion and accumulation of leukocytes in cerebral vessels are correlated with brain inflammation, which is a key feature of ECM. Concomitant with leukocyte aggregation, untreated mice exhibited evidence of vascular congestion characterized by increased RBC density within cerebral capillaries-a morphological manifestation of impaired microcirculation in experimental malaria. The treated groups effectively reduced the aggregation of inflammatory cells and alleviated this vascular congestion and associated increase in RBC density in brain capillaries (Fig 5i). The tissue-protective effects on liver, spleen and brain exhibited dose-dependent manner in both LL-37 and CRAMP groups.

Given the critical importance of exposure for efficacy, we evaluated the *in vivo* pharmacokinetics of LL-37/CRAMP. Plasma concentrations were measured 30 minutes after a single injection and 24 hours after the final dose of the 4-day regimen. At 30 minutes post-injection, peptide levels were significantly elevated in both uninfected and infected mice versus controls (*p* < 0.001), confirming successful delivery (S4a, S4b Fig). Notably, infected mice exhibited higher plasma CRAMP levels than uninfected (*p* < 0.01), suggesting infection may synergistically enhance CRAMP availability (S4a, S4b Fig). However, by 24 hours after the last dose, plasma CRAMP in infected mice was no longer distinguishable from baseline, and LL-37 was undetectable (S4c Fig). This indicates rapid *in vivo* degradation of the native peptides with minimal accumulation upon repeated dosing.

Based on the promising prophylactic results but considering their rapid clearance, we next assessed the peptides’ efficacy when treatment was initiated *after* infection establishment. A separate cohort received LL-37 or CRAMP (16 mg/kg/day for 4 days) only once parasitemia reached 8–15%. While this therapeutic regimen significantly reduced parasitemia and downregulated systemic pro-inflammatory cytokines (IL-1β, IL-6, TNF-α, IFN-γ) compared to saline controls, it failed to improve histopathological organ pathology in the liver, spleen, or brain, and did not extend host survival (S5a–S5h Fig). The lack of therapeutic benefit correlates with the pharmacokinetic data, suggesting that rapid peptide degradation prevents the accumulation of sufficient bioactive concentrations to reverse established tissue damage.

Collectively, these results demonstrate a critical distinction: prophylactic administration of LL-37/CRAMP effectively controls parasitemia, modulates inflammation, protects organs, and prolongs survival. However, their rapid *in vivo* clearance limits efficacy, and therapeutic intervention initiated after patent parasitemia, while reducing parasite burden and systemic inflammation, is insufficient to rescue established pathology or improve survival outcomes.

### 6. Endogenous Cathelicidin (CRAMP) is essential for parasite control

To determine the physiological contribution of endogenous cathelicidin in malaria infection, we employed Cramp knockout (KO) mice in our experimental model. The absence of functional CRAMP resulted in a significantly exacerbated disease course. On day 4 post-infection, Cramp

**Fig 6:**
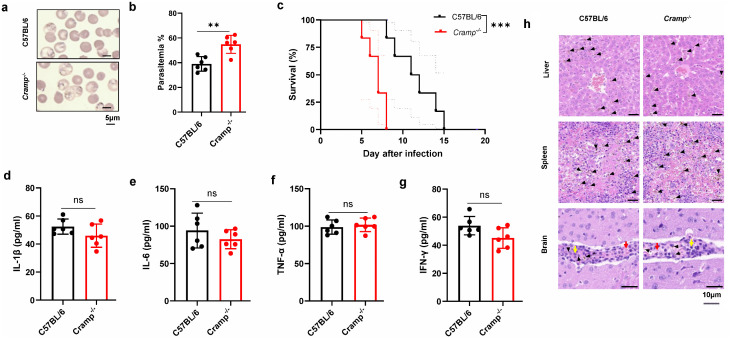
Endogenous cathelicidin (Cramp) is essential for parasite control. **(a)** Representative images of blood smears from C57BL/6 mice (day 4 post-infection), *Cramp*^*⁻/⁻*^ mice. Scale bar = 5 μm. **(b)** Quantification of parasitemia (day 4, n = 6 mice/group) in C57BL/6 mice, *Cramp*^*⁻/⁻*^ mice. **(c)** Survival curves (day 21, n = 6 mice/group) of C57BL/6 mice, *Cramp*^*⁻/⁻*^ mice after infection. **(d–g)** Quantification of plasma levels of IL-1β **(d)**, IL-6 **(e)**, TNF-α **(f)**, and IFN-γ **(g)** (day 4, n = 6 mice/group) in C57BL/6 mice, *Cramp*^*⁻/⁻*^ mice. **(h)** Representative H&E-stained sections of liver, spleen, and brain tissues (day 4, n = 3 mice/group) from C57BL/6 mice, *Cramp*^*⁻/⁻*^ mice. Black arrows point to malarial pigment; red arrows indicate the sequestration of RBCs; yellow arrows denote inflammatory cells; red boxes indicate RePu or WhPu. Scale bar = 10 μm. ns: not significant; **p < 0.01, ***p < 0.001 indicate statistically significant differences.

KO mice exhibited substantially higher parasitemia compared to wild-type (WT) controls (Fig 6a, 6b), indicating a severely impaired capacity to limit early parasite replication. This defect in controlling the rise of parasite burden directly translated to a worsened clinical outcome, as evidenced by a significant reduction in the survival time of KO mice (Fig 6c). These data establish that endogenous CRAMP plays a non-redundant and critical role in the host’s innate defense system against blood-stage *P. berghei* infection by limiting initial parasite expansion.

A notable and unexpected finding emerged from the analysis of the systemic inflammatory response. Despite the higher parasite burden, the absence of CRAMP did not lead to a corresponding increase in the plasma levels of key pro-inflammatory cytokines, including IL-1β, IL-6, TNF-α, and IFN-γ, which were measured at the same time point (Fig 6d-6g). The cytokine profiles of infected KO mice were not significantly different from those of their infected WT counterparts. This dissociation between parasite burden and systemic cytokine levels suggests that the primary role of endogenous CRAMP at this early stage of infection is direct parasite restriction rather than broad modulation of systemic inflammation. The absence of CRAMP leads to uncontrolled parasite replication, but this does not result in a proportionally amplified systemic cytokine response, indicating that the peptide’s essential function in host defense is mediated through its direct antiplasmodial activity. This suggests that the protective mechanism of endogenous CRAMP is independent of the modulation of these specific systemic inflammatory mediators during the early phase of infection. Consistent with the cytokine data, histopathological assessment at the defined endpoints also revealed no marked aggravation of tissue injury in Cramp KO mice relative to WT controls (Fig 6h). The degree of hemozoin deposition in the liver and inflammatory cell accumulation within cerebral vessels (a feature of vascular inflammation) was comparable between the two groups. This indicates that the increased mortality in KO mice is more directly attributable to their failure to restrain parasitemia rather than to a heightened immunopathological damage at the tissue level at the time points examined.

Collectively, these *in vivo* findings that impaired early parasite control without heightened systemic inflammation or tissue pathology converge with our *in vitro* evidence of direct lytic activity to demonstrate that endogenous CRAMP functions as a crucial first-line defender in malaria primarily by directly targeting and disrupting the infected red blood cell. This mechanism effectively delays the exponential rise in parasitemia and fatal outcome.

## Discussion

Our study establishes the human cathelicidin LL-37 as a selective antimalarial agent and elucidates the mechanism underlying its selective action against *Plasmodium falciparum*. While this potency is significantly lower than that of front-line drugs like artemisinin (typically in the nM range), we elucidate its distinct mechanism and demonstrate its physiological relevance during malaria infection.

Initial *in vitro* assays confirmed that LL-37 acts as a direct inhibitor of blood-stage *P. falciparum*, exhibiting low micromolar IC₅₀ values against drug-sensitive (3D7), chloroquine-resistant (Dd2), and artemisinin-resistant (803) strains. The comparable efficacy across these phenotypes indicates a mechanism distinct from conventional antimalarials, one that is not impaired by mutations in Kelch13 or Pfcrt, which are associated with artemisinin and chloroquine resistance, respectively [39–41]. We propose that this distinct mechanism involves a multi-stage interaction with the host erythrocyte membrane, which has been pathologically remodeled by the parasite. This interpretation is supported by the observation that even the isolated N-terminal (LE-16) or C-terminal (FR-13) fragments of LL-37 could achieve a partial (~40%) inhibition plateau at low concentrations, suggesting that initial membrane binding or perturbation is a shared, saturable function accessible to either domain alone. However, only the full-length LL-37, integrating both domains, demonstrates the capacity to surpass this threshold and achieve complete parasite clearance at higher concentrations. This underscores that LL-37’s primary target is not a parasite-encoded protein or metabolic pathway, but the host-derived erythrocyte membrane that has been pathologically remodeled by the parasite. This host-directed approach, which exploits a pathological alteration of the host cell membrane rather than targeting parasite-specific pathways, means that conventional resistance mechanisms are likely ineffective against LL-37. Therefore, the peptide’s activity is insusceptible to the existing drug resistance landscape, positioning it as a valuable template for developing novel antimalarials with a novel mode of action.

Further characterization revealed that LL-37 exhibits stage-dependent antimalarial activity, with the highest potency against schizont-infected red blood cells (iRBCs). This stage-specific susceptibility aligns with observed hemolysis patterns, where schizont-iRBCs were significantly more vulnerable to LL-37-mediated lysis than ring- or trophozoite-iRBCs or uninfected RBCs. Minimal hemolysis of uninfected RBCs even at high peptide concentrations indicates a favorable preliminary therapeutic window. Furthermore, the time-dependent inhibition across all stages emphasizes that sustained exposure is key to maximizing LL-37’s parasiticidal effect, providing preliminary insights for potential dosing strategies in future therapeutic development.

The mechanistic dissection revealed a dependency on the pathological membrane alterations induced by the parasite. Reconstitution experiments demonstrate that both cholesterol depletion and phosphatidylserine externalization can independently sensitize erythrocytes to LL-37-mediated hemolysis, as evidenced by the dose-dependent lytic enhancement triggered by either MβCD or A23187 in the presence of the peptide. Moreover, when subthreshold cholesterol depletion was combined with PS exposure, the combination dramatically potentiated LL-37’s lytic activity beyond the effect of either condition alone.

The physiological relevance of our *in vitro* findings is strongly supported by the significant upregulation of plasma LL-37/CRAMP observed in both human malaria patients and *P. berghei*-infected mice. However, the concentration and source of the peptide critically determine its predominant mechanism of action. In human patients, the elevated yet modest systemic levels of LL-37 (~7.3 nM) are orders of magnitude lower than the *in vitro* IC₅₀ for direct killing. This discrepancy underscores that the standard *in vitro* killing assay cannot fully capture the complexity of the host microenvironment, and points to a scenario where endogenously produced LL-37 likely operates through secondary, immunomodulatory functions (e.g., chemoattraction and immune cell activation) that are not modeled in simple parasiticidal assays. Notably, the low endogenous LL-37 concentrations in malaria patients do not refute the mechanistic insights from our *in vitro* analysis; instead, they reflect the concentration-dependent functional differentiation of LL-37. Our *in vitro* experiments form the core scientific foundation of this study: they identify the novel, host-directed lytic mechanism of LL-37 against iRBCs—an innovation that bypasses antimalarial drug resistance—and provide critical parameters (effective concentration, structural specificity, stage tropism) for exogenous therapeutic administration. This *in vitro* work is also the essential control for dissecting LL-37’s dual *in vivo* effects, allowing us to distinguish direct iRBC lysis (the primary driver of therapeutic efficacy) from secondary immunomodulation (the dominant role at physiologic endogenous concentrations). Without this *in vitro* dissection, the rational translation of LL-37 into an antimalarial therapy would not be possible.

This model is complemented and extended by our *in vivo* prophylactic and therapeutic intervention studies, as well as genetic studies in mice. Exogenous prophylactic administration of both human LL-37 and its murine ortholog CRAMP conferred significant protection benefits in the *P. berghei* mouse model by engaging a dual mechanism: direct parasite suppression coupled with profound attenuation of immunopathology as evidenced by reduced pro-inflammatory cytokines and tissue damage. The functional importance of endogenous cathelicidin was demonstrated using CRAMP knockout (KO) mice. The increased parasitemia and reduced survival in KO mice compared to infected controls establish CRAMP as an essential component of the host’s innate defense against blood-stage *P. berghei* infection. Interestingly, the absence of CRAMP did not significantly alter cytokine levels or histopathology severity compared to infected WT mice at the examined endpoint. The absence of systemic cytokine differences between WT and Cramp-KO mice underscores that endogenous CRAMP is not a primary regulator of malaria-associated proinflammatory cytokine production. Its core function is the direct lysis of early-stage iRBCs to slow parasite replication—a mechanism critical for host survival (KO mice have reduced survival) but one that does not alter the systemic immune signaling cascade. Any subtle immunomodulatory effects of CRAMP are likely tissue-localized (undetectable in plasma) or require a larger parasitemia difference than that observed at the day 4 sampling point to drive measurable systemic cytokine changes. Similarly, the reduced pro-inflammatory cytokine levels observed in peptide-treated mice may be partially attributable to the direct suppression of parasitemia, rather than a direct immunomodulatory effect of LL-37/CRAMP. These effects are poorly replicated in standard *in vitro* systems that lack integrated immune circuits and long-term exposure dynamics.

It should be noted as a limitation of the current study that the assessment of malaria-associated organ pathology (e.g., hemozoin deposition, tissue architectural changes) was primarily based on subjective, qualitative observations of H&E-stained sections, rather than quantitative morphometric analysis or automated image quantification. While we standardized the sectioning and staining protocols and conducted blinded evaluations to minimize bias, this qualitative approach inherently limits the precision and reproducibility of the pathological assessment.

The contrast between the efficacy of prophylactic LL-37/CRAMP administration and the limited therapeutic benefit in established infection provides critical insights into the peptide’s anti-malaria mechanism and translational constraints. In the prophylactic setting, early 4-day dosing (initiated before high parasitemia) not only suppressed parasite replication and systemic inflammation but also ameliorated liver, spleen, and brain pathology, and significantly prolonged host survival. This protective effect stems from the peptide’s ability to act upstream of irreversible tissue damage: by inhibiting schizont maturation and reducing pro-inflammatory cytokine release at the initial stage of infection, 16 mg/kg LL-37/CRAMP prevents the onset of key pathological events, including hemozoin deposition, splenic architectural remodeling, and cerebral microvascular congestion. In contrast, therapeutic intervention with the same 16 mg/kg dose (initiated at 8–15% parasitemia) only reduced parasitemia and systemic cytokine levels, but failed to improve organ pathology or extend survival. Two core factors underpin this discrepancy. First, pharmacokinetic limitations of native peptides: the short plasma half-life of LL-37/CRAMP (due to rapid degradation by serum proteases) means that even 4-day consecutive dosing cannot maintain sustained therapeutic concentrations *in vivo*. In established infection, the large and tissue-sequestered parasite population rapidly rebounds after transient suppression, re-initiating inflammatory damage that negates any temporary benefit. Second, failure to target localized tissue-resident inflammatory pathways: by the time parasitemia reaches 8–15%, tissue damage is no longer driven solely by systemic cytokines—it is sustained by localized inflammatory circuits, such as hepatic stellate cell activation, splenic macrophage infiltration, and cerebral vascular endothelial dysfunction. Systemically administered LL-37/CRAMP cannot efficiently penetrate tissue microenvironments or disrupt these localized pathways, even though it reduces circulating cytokine levels. This explains why therapeutic treatment fails to reverse established pathology, whereas prophylactic treatment prevents it from occurring in the first place.

The plasma concentration data further clarify the pharmacokinetic constraints underlying the divergent efficacy of LL-37/CRAMP in prophylactic vs. therapeutic settings. The significant elevation of peptide levels at 30 min post-injection confirms that exogenous LL-37/CRAMP can achieve biologically relevant concentrations *in vivo*, which supports the direct antiplasmodial and immunomodulatory effects observed in the 4-day prophylactic assay. The higher CRAMP levels in infected mice at this time point also align with our result of infection-induced endogenous CRAMP upregulation, suggesting a synergistic interaction between exogenous peptide administration and host innate immune activation that may contribute to the prophylactic efficacy. However, the minimal drug accumulation and extremely low LL-37 levels detected at 24 h post-final dose highlight the severe *in vivo* degradation of native peptides. Even 4 days of consecutive dosing failed to maintain sustained therapeutic concentrations, a critical pharmacokinetic limitation that explains why prolonged peptide exposure is required to maximize antiplasmodial activity *in vitro*, but difficult to achieve *in vivo*. This rapid degradation not only limits the efficacy of prophylactic treatment (where only survival prolongation, but not complete cure, is observed) but also exacerbates the failure of therapeutic intervention in established infection: the short-lived peptide levels cannot sustain sufficient exposure to eliminate tissue-sequestered parasites or reverse localized inflammation, even at the highest tested dose.

Our *in vitro* data showed a minimal 3–5% lysis of uninfected RBCs (Fig 3a), which raised questions about *in vivo* safety. However, our *in vivo* hemolysis assay demonstrated that LL-37/CRAMP administration at therapeutic doses does not induce detectable plasma hemoglobin elevation (S3 Fig). This discrepancy may be explained by the presence of plasma proteins (e.g., albumin) *in vivo* that bind to LL-37/CRAMP and reduce their off-target interaction with uninfected RBC membranes. Additionally, the preferential targeting of infected RBCs by LL-37/CRAMP, mediated by parasite-induced membrane alterations (e.g., PS externalization, cholesterol depletion), further minimizes off-target hemolytic effects. Collectively, these data support the safety profile of LL-37/CRAMP as anti-malarial agents with negligible detrimental hemolytic effects in *vivo*. Notably, free plasma hemoglobin is a relatively insensitive marker of *in vivo* hemolysis, as host scavenger systems—most notably haptoglobin—rapidly clear free hemoglobin from the circulation until these homeostatic pathways are overwhelmed. While the absence of elevated free hemoglobin is a reassuring safety finding, a more comprehensive assessment of hemolysis and associated tissue injury would ideally include additional classic biomarkers such as lactate dehydrogenase (LDH), alanine transaminase (ALT), and hematocrit, which we recommend for future studies to further validate the *in vivo* safety profile of LL-37/CRAMP. An additional advantage of LL-37 as a potential therapeutic candidate lies in its established safety profile and clinical translatability. The peptide has advanced to human trials, as demonstrated in a Phase I/II clinical study conducted by the MD Anderson Cancer Center, which evaluated intratumoral injection of LL-37 in patients with melanoma (ClinicalTrials.gov Identifier: NCT02225366) [42]. The progression of LL-37 to this clinical stage provides indirect yet compelling evidence of its acceptable safety and tolerability in humans. This clinical precedent, combined with our observations of selective iRBC lysis and minimal off-target toxicity, supports the translational feasibility of exploring LL-37-based antimalarial strategies.

The promising *in vitro* and *in vivo* efficacy and the unique mechanism of LL-37 must be balanced against translational challenges inherent to this peptide class, which are particularly acute in the context of malaria field treatment. As noted, the need for intravenous administration and the failure to achieve complete cure in our murine model, which resulted in delayed mortality, directly reflect LL-37’s susceptibility to serum proteases and its consequently short plasma half-life. A notable limitation of our *in vivo* pharmacokinetic analysis of LL-37 and CRAMP is the lack of intermediate sampling time points between 30 min and 24 h post-administration, which precludes precise quantification of the rate and kinetics of native peptide degradation in murine plasma during malaria infection. While our data confirm rapid clearance of heterologous LL-37 to undetectable levels by 24 h and stable CRAMP concentrations driven by infection-induced endogenous induction, the absence of time points such as 1, 6, or 12 h means we cannot define the exact plasma half-life of either peptide, nor identify the key time window during which bioactive concentrations are maintained *in vivo*. This gap limits a full understanding of the relationship between peptide exposure duration and antiplasmodial efficacy, a critical parameter for optimizing dosing regimens of LL-37/CRAMP or their stabilized analogs in future studies. This pharmacokinetic profile hinders the maintenance of therapeutic concentrations. Furthermore, the high synthesis cost of full-length LL-37 and its potential cytotoxicity toward human cells at elevated doses, as highlighted in the literature, pose additional barriers to its development as a safe, affordable, and scalable field therapy. Its stage-specific potency against sequestered schizonts presents a dual aspect: while it targets a pathology-critical stage, effective delivery to tissue-resident parasites poses an additional pharmacokinetic hurdle. These limitations underscore that native LL-37 is not an optimal standalone therapeutic agent, but rather a compelling lead compound. They are further highlighted by our therapeutic intervention data: even at the highest tested dose, native LL-37/CRAMP cannot rescue established malaria pathology or improve survival, underscoring that optimizing peptide pharmacokinetics and tissue penetration is essential for translational success. Overcoming these barriers represents a clear path for development: engineering stabilized, cost-effective LL-37 analogs (e.g., using D-amino acids, cyclization, or truncated minimal motifs to reduce synthetic cost and proteolysis), incorporating these analogs into affordable, long-acting delivery systems (e.g., depot formulations or targeted nanocarriers) to enhance stability, reduce dosing frequency, and mitigate toxicity, or utilizing LL-37 derivatives as a component in combination therapies could collectively address the issues of cost, stability, toxicity, and efficacy, fully exploiting its dual antiparasitic and immunomodulatory potential in a practicable manner.

In conclusion, this study establishes LL-37 as a selective, and physiologically relevant antimalarial effector. Its activity is maximized by extended exposure and preferentially targets schizont-infected RBCs by exploiting their pathologically depleted membrane cholesterol, with PS externalization playing a synergistic role. The significant upregulation during infection and the notable *in vivo* efficacy of exogenous LL-37/CRAMP in prophylactic settings, encompassing direct parasite inhibition and attenuation of malaria-associated pathology, support the role of cathelicidins as key components of the host defense against malaria and as lead components of therapeutic candidates. Although LL-37 shows significant antiplasmodial effects *in vitro* and *in vivo*, the effective concentration *in vitro* (IC₅₀ ≈ 5μM) is relatively high, and intravenous injection in mice can only prolong survival rather than achieving complete cure. More importantly, therapeutic administration of LL-37/CRAMP in established malaria fails to ameliorate organ pathology or extend survival, which may be related to the short half-life of endogenous peptides in vivo and their inability to target localized tissue-resident inflammatory pathways. Future research should therefore focus on translating this proof-of-concept by addressing the aforementioned pharmacokinetic and delivery challenges. Key directions include: (1) Developing stabilized LL-37 analogs or mimetics resistant to proteolysis; (2) Employing formulation or delivery strategies (e.g., nanocarriers) to enhance plasma half-life and target sequestered parasites; (3) Exploring combination therapies with existing antimalarials; and (4) Further dissecting the immunomodulatory pathways engaged by therapeutic cathelicidins. (5) Evaluating the efficacy of stabilized LL-37/CRAMP analogs or targeted delivery formulations in therapeutic intervention assays for established malaria, and exploring adjunctive therapy with front-line antimalarials (e.g., artemisinin derivatives) to synergistically enhance parasite clearance and tissue repair. Harnessing the dual anti-parasitic and host-protective functions of cathelicidins represents a promising exploratory strategy for combating malaria and its associated immunopathology.

## Supporting information

S1 Fig Validation of cholesterol repletion, phosphatidylserine (PS) binding competition, and LL-37 displacement by DOTAP. (a) Membrane cholesterol levels in *Plasmodium*-infected erythrocytes (iRBCs) after treatment with MβCD-cholesterol complexes at ratios of 1:80, 1:40, 1:20, relative to the infected control group. Data are mean ± SEM (n = 3). Statistical analysis: one-way ANOVA with Tukey’s multiple comparison test, *p < 0.05, **p < 0.01, ***p < 0.001 vs. infected control. (b) Representative flow cytometry dot plots (Annexin V vs. Hoechst 33258) showing PS accessibility in iRBCs (Control), LL-37 (5 μM), or DOTAP (20 μM). Reduced Annexin V positivity (Q2 quadrant) indicates PS binding by LL-37 or DOTAP. (c) Quantification of Annexin V positivity in iRBCs from (b). Data are mean ± SEM (n = 3). Statistical analysis: one-way ANOVA with Tukey’s multiple comparison test, ***p < 0.001 vs. Control. (d) Concentration-dependent increase in supernatant LL-37 levels after co-incubation with DOTAP (0, 5, 10, 20 μM), reflecting displacement of LL-37 from PS-exposed iRBC membranes. Data are mean ± SEM (n = 3). Statistical analysis: one-way ANOVA with Tukey’s multiple comparison test, *p < 0.05, **p < 0.01, ***p < 0.001 vs. 0 μM DOTAP group.
(TIF)

S2 Fig Dynamic changes in reticulocyte-malaria related clinical score (RMCBS) in *P. berghei*-infected mice following treatment with LL-37 or CRAMP. Mice were infected with *P. berghei* ANKA and treated intravenously with LL-37 or CRAMP at doses of 1, 4, or 16 mg/kg/day for 4 consecutive days. The non-treated control group (NS) received saline. The RMCBS score (range 0–12) was monitored daily for 20 days post-infection to assess disease severity. Each symbol represents an individual mouse (n = 6 per group).
(TIF)

S3 Fig Hemolytic activity of LL-37 and CRAMP in uninfected and *Plasmodium*-infected erythrocytes. Hemolysis (%) was quantified in uninfected (uninfect) and *Plasmodium*-infected (infect) erythrocytes after treatment with LL-37 or CRAMP. Data are presented as mean ± SEM (n = 4), with individual data points shown. All treatment groups exhibited minimal hemolysis (<0.5%), indicating negligible off-target erythrocyte toxicity at therapeutic peptide concentrations.
(TIF)

S4 Fig *In vivo* plasma concentrations of CRAMP and LL-37 in uninfected and *Plasmodium*-infected mice. (a, b) Plasma concentrations of CRAMP (a) and LL-37 (b) were measured 30 minutes after a single intravenous injection in uninfected (*uninfect/N*) or *Plasmodium*-infected (*infect/N*) mice, with or without CRAMP (a)/LL-37 (b) treatment. Data are mean ± SEM (n = 4–6 mice per group). Statistical analysis: one-way ANOVA with Tukey’s multiple comparison test, *p < 0.05, *p < 0.01, ***p < 0.001. (c) Plasma concentrations of LL-37 or CRAMP after 4 consecutive days of intravenous administration in uninfected and *Plasmodium*-infected mice. No significant difference (ns) was observed between uninfected and infected mice following prolonged treatment. Data are mean ± SEM (n = 4–6 mice per group).
(TIF)

S5 Fig Therapeutic efficacy of LL-37, CRAMP, and chloroquine (CQ) in a *P. berghei* ANKA malaria model. (a, b) Parasitemia kinetics (a) and day-4 parasitemia quantification (b) in *P. berghei*-infected mice treated with LL-37 (16 mg/kg/day), CRAMP (16 mg/kg/day), CQ (positive control), or saline (NS, negative control). Data are mean ± SEM (n = 6 per group). Statistical analysis: one-way ANOVA with Tukey’s multiple comparison test, **p < 0.01, ***p < 0.001. (c) Survival curves of infected mice over 12 days post-infection. Statistical analysis: Log-rank (Mantel-Cox) test, **p < 0.01, ***p < 0.001 vs. NS group. (d-g) Plasma levels of pro-inflammatory cytokines IL-6 (d), IL-1β (e), TNF-α (f), and IFN-γ (g) in infected mice 4 days post-infection. Data are mean ± SEM (n = 6 per group). Statistical analysis: one-way ANOVA with Tukey’s multiple comparison test, *p < 0.05, **p < 0.01, ***p < 0.001. (h) Histopathological analysis of liver, spleen, and brain tissues from infected mice 4 days post-infection, stained with hematoxylin and eosin (H&E). Scale bars: 20 μm.
(TIF)

S1 Data Excel file containing the numerical data used to generate all the Figs. (RAR)
