# Supplementary figures and images for: LL-37 selectively targets Plasmodium-infected erythrocytes and exhibits antimalarial activity
Source: PLoS Pathog. 2026 Mar 17;22(3):e1014062. doi: 10.1371/journal.ppat.1014062 (PMC13004495; doi:10.1371/journal.ppat.1014062)

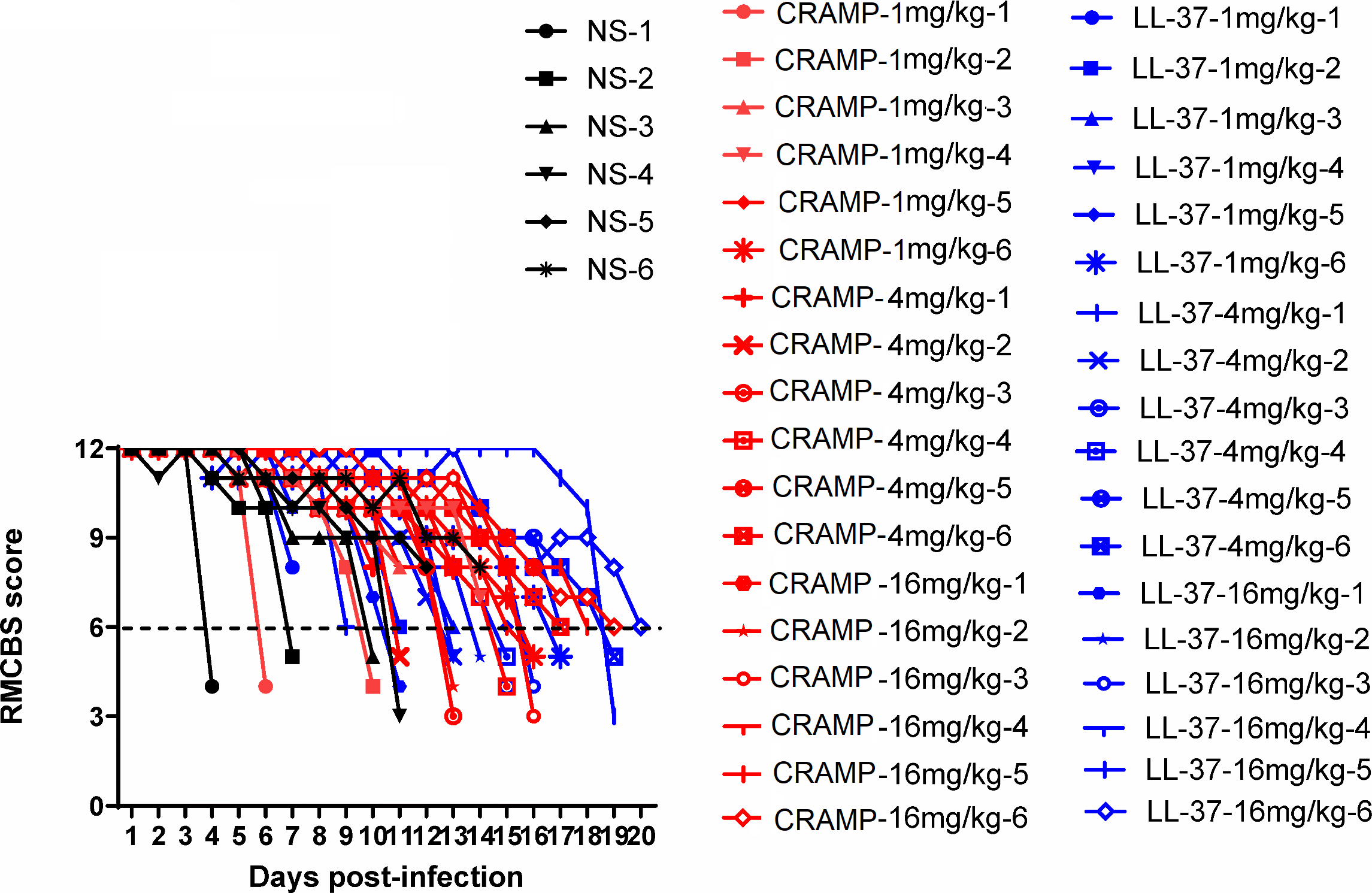

Supplement: S2 Fig — Mice were infected with P. berghei ANKA and treated intravenously with LL-37 or CRAMP at doses of 1, 4, or 16 mg/kg/day for 4 consecutive days. The non-treated control group (NS) received saline. The RMCBS score (range 0–12) was monitored daily for 20 days post-infection to assess disease severity. Each symbol represents an individual mouse (n = 6 per group). (TIF) [file ppat.1014062.s002.tif]

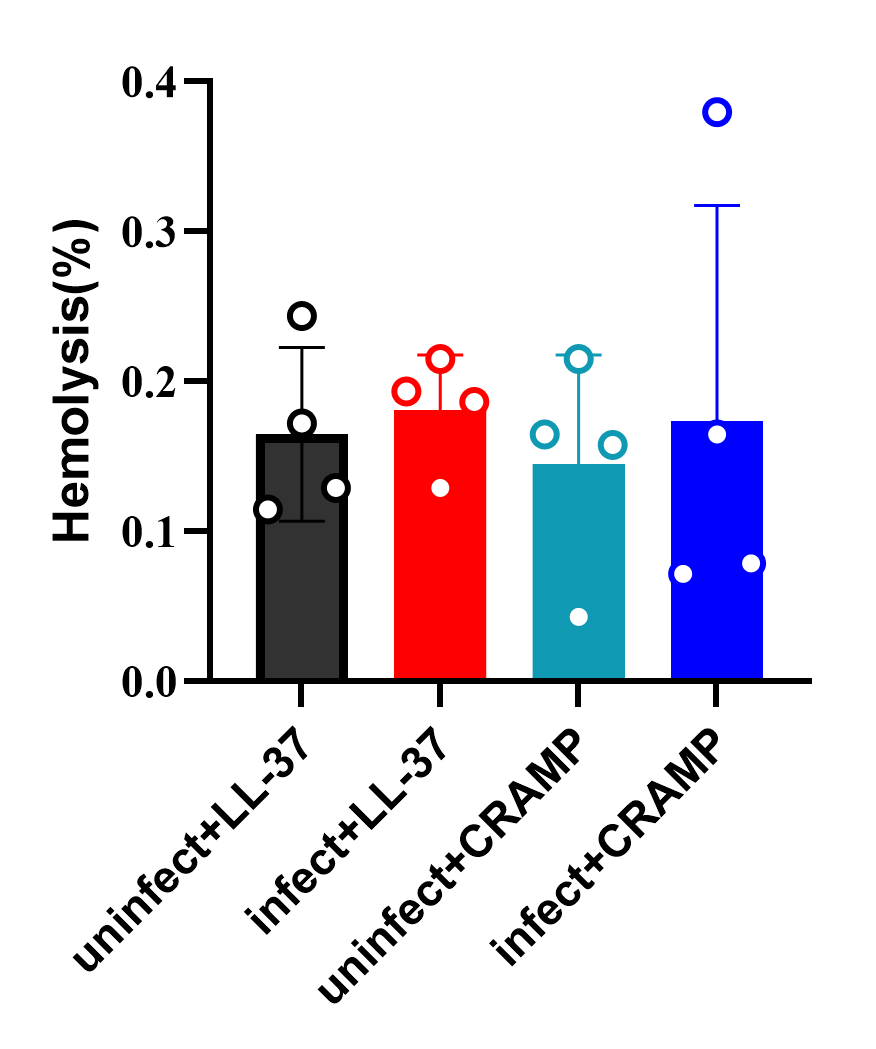

Supplement: S3 Fig — Hemolysis (%) was quantified in uninfected (uninfect) and Plasmodium-infected (infect) erythrocytes after treatment with LL-37 or CRAMP. Data are presented as mean ± SEM (n = 4), with individual data points shown. All treatment groups exhibited minimal hemolysis (<0.5%), indicating negligible off-target erythrocyte toxicity at therapeutic peptide concentrations. (TIF) [file ppat.1014062.s003.tif]

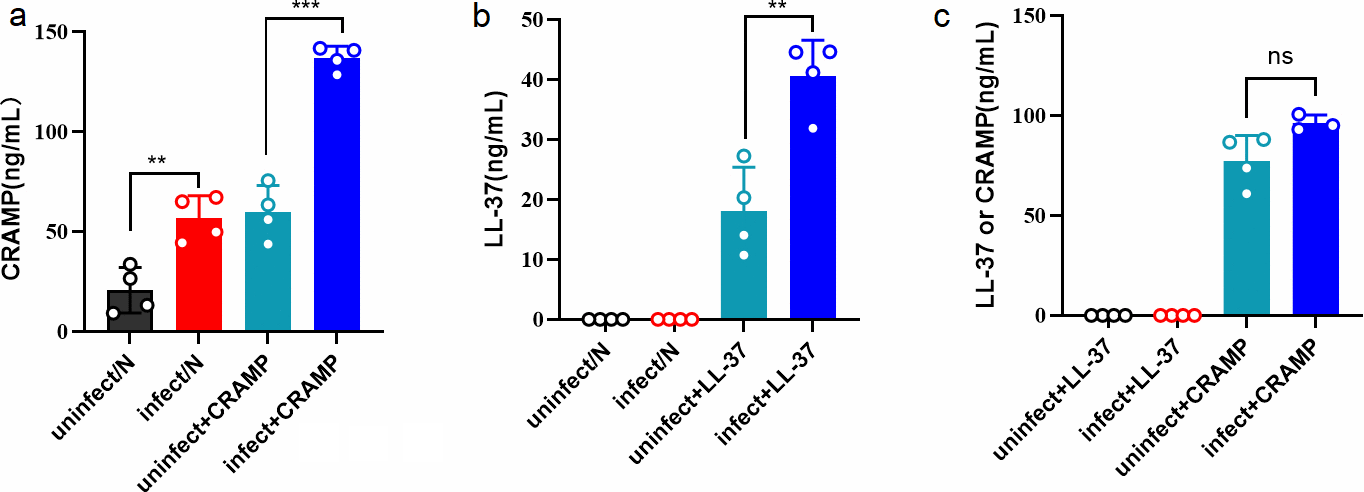

Supplement: S4 Fig — (a, b) Plasma concentrations of CRAMP (a) and LL-37 (b) were measured 30 minutes after a single intravenous injection in uninfected (uninfect/N) or Plasmodium-infected (infect/N) mice, with or without CRAMP (a)/LL-37 (b) treatment. Data are mean ± SEM (n = 4–6 mice per group). Statistical analysis: one-way ANOVA with Tukey’s multiple comparison test, *p < 0.05, *p < 0.01, ***p < 0.001. (c) Plasma concentrations of LL-37 or CRAMP after 4 consecutive days of intravenous administration in uninfected and Plasmodium-infected mice. No significant difference (ns) was observed between uninfected and infected mice following prolonged treatment. Data are mean ± SEM (n = 4–6 mice per group). (TIF) [file ppat.1014062.s004.tif]

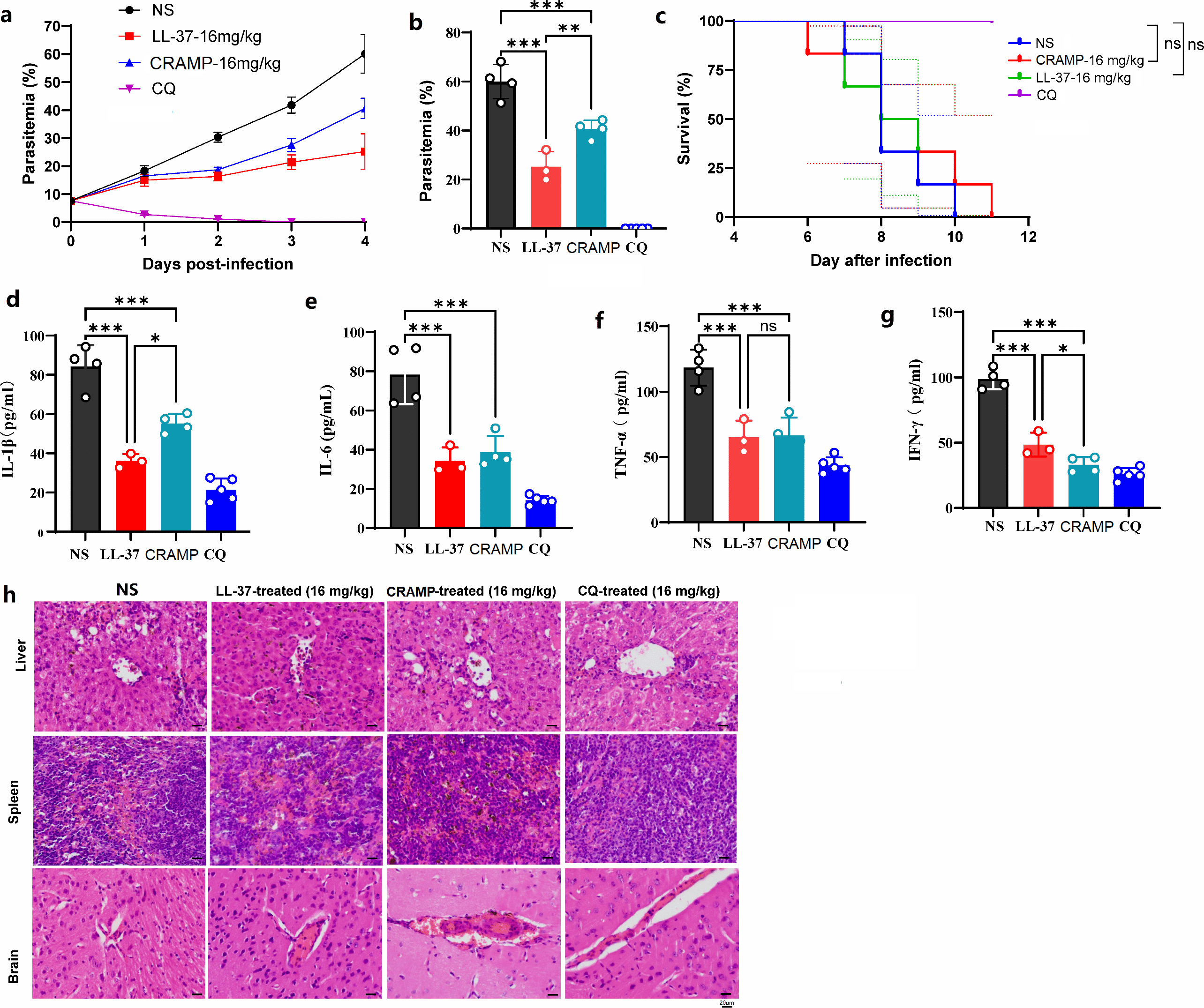

Supplement: S5 Fig — (a, b) Parasitemia kinetics (a) and day-4 parasitemia quantification (b) in P. berghei-infected mice treated with LL-37 (16 mg/kg/day), CRAMP (16 mg/kg/day), CQ (positive control), or saline (NS, negative control). Data are mean ± SEM (n = 6 per group). Statistical analysis: one-way ANOVA with Tukey’s multiple comparison test, **p < 0.01, ***p < 0.001. (c) Survival curves of infected mice over 12 days post-infection. Statistical analysis: Log-rank (Mantel-Cox) test, **p < 0.01, ***p < 0.001 vs. NS group. (d-g) Plasma levels of pro-inflammatory cytokines IL-6 (d), IL-1β (e), TNF-α (f), and IFN-γ (g) in infected mice 4 days post-infection. Data are mean ± SEM (n = 6 per group). Statistical analysis: one-way ANOVA with Tukey’s multiple comparison test, *p < 0.05, **p < 0.01, ***p < 0.001. (h) Histopathological analysis of liver, spleen, and brain tissues from infected mice 4 days post-infection, stained with hematoxylin and eosin (H&E). Scale bars: 20 μm. (TIF) [file ppat.1014062.s005.tif]
